# Phenotypic and Transcriptomic Characterization of Host-Associated Responses in a Carbapenem-Resistant *Klebsiella pneumoniae* ST11 Clinical Isolate

**DOI:** 10.3390/pathogens15030282

**Published:** 2026-03-05

**Authors:** Miaomiao Hua, Jun Ji, Zhongyu Wang, Jingjing Li, Xiaoli Cao

**Affiliations:** 1 Department of Laboratory Medicine, Nanjing Drum Tower Hospital, The Affiliated Hospital of Nanjing University Medical School, Nanjing 210008, China; huamiaomiao5432@163.com (M.H.); wzy740655@163.com (Z.W.); 2 Department of Laboratory Medicine, Nanjing Drum Tower Hospital Clinical College of Nanjing University of Chinese Medicine, Nanjing 210008, China; jijun0308@163.com

**Keywords:** *Klebsiella pneumoniae*, ST11 clone, carbapenem resistance, host adaptation, bacterial metabolism, iron acquisition, transcriptome, *fec* operon

## Abstract

Carbapenem-resistant *Klebsiella pneumoniae* (CRKP) sequence type 11 (ST11) is widely reported in Asia and represents an important clinical concern. Although antimicrobial resistance in ST11 isolates has been extensively investigated, less is known about the phenotypic characteristics and host-associated transcriptional responses of individual clinical isolates. In this study, we performed an in-depth characterization of a clinical ST11 CRKP isolate, NJGLYY4165. Phenotypic assays were used to evaluate bacterial growth, biofilm formation, serum resistance, inflammatory responses, and virulence in a *Galleria mellonella* infection model. Whole-genome sequencing and bacterial transcriptomic profiling were conducted to examine gene expression changes at 4 h during interaction with human intestinal epithelial cells and macrophages. NJGLYY4165 exhibited increased survival following exposure to normal human serum in vitro but displayed reduced lethality in the *Galleria mellonella* infection model compared with selected reference strains. Infection of host cells induced marked inflammatory responses. Transcriptomic analysis revealed extensive gene expression remodeling at the examined time point during host interaction, including upregulation of central metabolic pathways and iron acquisition systems, particularly under macrophage-associated conditions. This study provides a detailed phenotypic and single-time-point transcriptomic description of host-associated responses in a clinical ST11 CRKP isolate. The observed metabolic and iron acquisition responses may contribute to adaptation under host-associated conditions; however, because comparative analyses with non-ST11 or non-epidemic strains were not included, it remains unclear whether these features are isolate-specific, ST11-associated, or represent general adaptive responses of *K. pneumoniae*. Further comparative and time-course studies across diverse clinical isolates will be required to clarify temporal dynamics and broader epidemiological relevance.

## 1. Introduction

The global dissemination of carbapenem-resistant *Klebsiella pneumoniae* (CRKP) has become a major public health challenge, severely limiting therapeutic options and contributing to increased morbidity and mortality in healthcare settings [1]. Among the diverse CRKP lineages, sequence type 11 (ST11) isolates have emerged as the predominant epidemic group in many Asian countries, particularly in China [2]. These ST11 isolates are frequently associated with multidrug resistance and have been implicated in hospital outbreaks [3]. In some instances, ST11 backgrounds have been linked to the convergence of antimicrobial resistance and virulence determinants [4]. Despite their epidemiological importance, detailed phenotypic and transcriptional characterizations of individual clinical ST11 CRKP isolates during host interaction remain limited.

The pathogenicity of *K. pneumoniae* is multifactorial and involves colonization, evasion of innate immune defenses, resistance to complement-mediated killing, survival within phagocytic cells, and biofilm formation [5,6]. While these traits have been extensively investigated at the species level, less is known about how individual epidemic ST11 isolates respond at the transcriptional level during host-associated conditions. In addition to classical virulence determinants, bacterial fitness during infection may be influenced by stress tolerance and metabolic responses to host-imposed pressures [7,8].

Host-associated environments expose bacteria to nutrient limitation, oxidative stress, and metal ion sequestration [9]. Transcriptomic analyses during host–pathogen interactions provide a useful approach for describing bacterial gene expression patterns under such conditions [10,11]. However, transcriptomic studies focusing specifically on clinical ST11 CRKP isolates remain relatively scarce.

In the present study, we performed a detailed phenotypic and transcriptomic characterization of a clinical ST11 CRKP isolate, NJGLYY4165, identified during hospital surveillance and exhibiting notable serum survival. Rather than aiming to define lineage-wide characteristics, this work focuses on describing the phenotypic traits and single-time-point transcriptional responses of this isolate during interaction with human intestinal epithelial cells and macrophages. We compared NJGLYY4165 with selected reference strains to evaluate growth, biofilm formation, serum resistance, inflammatory responses, and virulence in the *Galleria mellonella* model. We then performed RNA sequencing at 4 h post-infection to characterize gene expression changes under host-associated conditions. This study provides a descriptive framework for understanding host-associated responses in a clinical ST11 isolate and may inform future comparative and time-course investigations involving larger strain collections.

## 2. Materials and Methods

### 2.1. Bacterial Strains and Growth Conditions

The primary isolate investigated in this study was NJGLYY4165, a clinical CRKP ST11 isolate obtained in 2015 from a hospitalized patient at our institution during routine surveillance. The isolate has been preserved in our laboratory collection and has been detected in previous regional epidemiological investigations [12,13]. For comparative phenotypic analyses, five well-characterized reference strains—ATCC BAA-1705, ATCC BAA-1706, ATCC 2146, ATCC 2524, and ATCC 13439—were purchased from the American Type Culture Collection (ATCC, Manassas, VA, USA). All strains were identified using matrix-assisted laser desorption/ionization time-of-flight mass spectrometry (MALDI-TOF MS; BioMérieux, Marcy-l’Étoile, France), using the manufacturer’s standard protocol and database (version 3.2). Bacterial stocks were stored at −80 °C in glycerol-containing cryoprotectant. Prior to experiments, strains were cultured on MacConkey agar (Oxoid, Altrincham, UK) or in lysogeny broth (LB; BD Difco, Franklin Lakes, NJ, USA) at 37 °C with shaking (200 rpm), unless otherwise stated.

### 2.2. Whole-Genome Sequencing and In Silico Analysis

Genomic DNA of NJGLYY4165 was extracted using a commercial bacterial genomic DNA extraction kit (Tiangen Biochemical Technology Co., Ltd., Beijing, China) according to the manufacturer’s instructions. Whole-genome sequencing (WGS) was performed by Tiangen Biochemical Technology Co., Ltd. using an Illumina sequencing platform. Raw sequencing reads were quality filtered and de novo assembled using CLC Genomics Workbench software (version 21.0.4, QIAGEN, Kastrup, Denmark). Genome annotation and in silico analyses, including multilocus sequence typing (MLST), antimicrobial resistance gene identification, and virulence factor prediction, were conducted using online tools provided by the Center for Genomic Epidemiology (http://www.genomicepidemiology.org/, accessed on 12 June 2024).

### 2.3. Phenotypic Characterization

#### 2.3.1. Growth Curve Assay

Overnight bacterial cultures were diluted 1:100 in fresh LB medium and transferred into 96-well microplates. Bacterial growth was monitored by measuring optical density at 620 nm (OD_620_) every hour for 7 h using a microplate reader (BioTek, Winooski, VT, USA). Experiments were performed in triplicate [14].

#### 2.3.2. Biofilm Formation Assay

Biofilm formation was quantified using a crystal violet staining method as previously described [15]. Briefly, bacterial suspensions were inoculated into 96-well polystyrene plates and incubated at 37 °C for 24 h. After washing with phosphate-buffered saline (PBS), adherent biofilms were stained with 0.1% crystal violet for 15 min. The dye was solubilized with 30% glacial acetic acid, and absorbance was measured at 550 nm (OD_550_).

#### 2.3.3. Serum Killing Assay

Bacterial serum resistance was assessed as described previously [16]. Briefly, bacteria were harvested at mid-log phase, washed, and resuspended in PBS. Bacterial suspensions were mixed with pooled normal human serum (Innovative Research, Plymouth, MN, USA) at a ratio of 1:3 and incubated at 37 °C. Samples were collected at 0, 1, 2, and 3 h, serially diluted, and plated on LB agar for colony counting. Survival rates were calculated as (CFU at time t/CFU at time 0) × 100%. All assays were performed with at least three independent biological replicates

### 2.4. Cell Culture and Infection Models

Human intestinal epithelial cells (Int407; ATCC CCL-6) and murine macrophages (J774A.1; ATCC TIB-67) were cultured in Eagle’s Minimum Essential Medium (EMEM) or Dulbecco’s Modified Eagle Medium (DMEM), respectively, supplemented with 10% fetal bovine serum (Gibco, Brooklyn, NY, USA), at 37 °C in a humidified atmosphere containing 5% CO_2_.

For adhesion assays [17], Int407 cells were infected with bacteria at a multiplicity of infection (MOI) of 50 and incubated for 4 h. For macrophage internalization assays [17], J774A.1 cells were infected at an MOI of 50 for 1 h, followed by treatment with hygromycin (Sigma-Aldrich, St. Louis, MO, USA) to eliminate extracellular bacteria. Host cells were then lysed with 0.2% Triton X-100, and intracellular bacteria were quantified by plating serial dilutions.

For cytokine analysis, infected cell culture supernatants were collected, and interleukin-6 (IL-6) and interleukin-8 (IL-8) levels were measured using commercial ELISA kits (R&D Systems, Minneapolis, MN, USA), according to the manufacturer’s instructions.

### 2.5. Galleria mellonella Infection Model

The virulence of NJGLYY4165 and reference strains was evaluated using the *Galleria mellonella larvae* infection model, a widely used surrogate system for assessing bacterial pathogenicity and innate immune interactions, as previously described [18]. Groups of ten *larvae* (approximately 250 mg each) were injected with 10 μL of bacterial suspensions containing 10^4^–10^7^ CFU/mL via the last proleg. Control *larvae* were injected with sterile PBS. *Larvae* were incubated at 37 °C in the dark, and survival was recorded every 12 h for up to 72 h. Death was defined as the absence of movement in response to tactile stimulation combined with melanization. Although this invertebrate model does not reproduce complement-dependent systemic infection or adaptive immune responses, it provides a comparative system for assessing virulence-associated phenotypes under standardized conditions.

### 2.6. Bacterial Transcriptome Sequencing and Analysis

To examine bacterial gene expression under host-associated conditions, Int407 and J774A.1 cells were infected with NJGLYY4165 at an MOI of 50 and incubated for 4 h. The 4 h time point was selected to allow sufficient bacterial recovery and preservation of RNA integrity while maintaining host cell viability. This time point is commonly used in host–pathogen interaction studies to characterize bacterial gene expression under host-associated conditions [19].

Following co-culture, bacteria were recovered, and total RNA was extracted. RNA quality was assessed prior to library preparation using the NEBNext^®^ Ultra™ RNA Library Prep Kit (New England Biolabs, Ipswich, MA, USA). Libraries were sequenced on an Illumina HiSeq 2000 platform to generate 100 bp paired-end reads.

Raw reads were processed using Trimmomatic and mapped to the NJGLYY4165 reference genome using Bowtie2 (v2.4.5). Gene expression quantification and differential expression analysis were performed using DESeq2 (v1.30.1). Genes with an adjusted *p*-value (padj) < 0.05 and |log_2_ fold change| > 1 were considered significantly differentially expressed. Functional enrichment analyses were conducted using Gene Ontology (GO) and Kyoto Encyclopedia of Genes and Genomes (KEGG) databases.

### 2.7. Statistical Analysis

All experiments were performed with at least three independent biological replicates. Data are presented as mean ± standard deviation (SD). Statistical analyses were performed using GraphPad Prism software (version 10.1.2, GraphPad Software, Boston, MA, USA). Differences between groups were evaluated using Student’s *t*-test or one-way analysis of variance (ANOVA) with appropriate post hoc tests. A *p*-value < 0.05 was considered statistically significant.

## 3. Results

### 3.1. NJGLYY4165 Exhibits Strong Complement Resistance but Does Not Display Enhanced Virulence in the Galleria mellonella Model

To characterize the phenotypic profile of the ST11 isolate NJGLYY4165, we compared its growth kinetics, biofilm formation, serum resistance, and in vivo virulence with a panel of reference strains.

**Figure 1:**
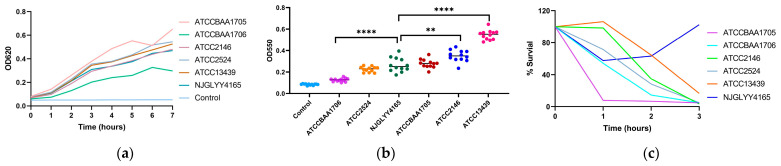
Comparative Analysis of Growth curve, Biofilm Formation, and Serum Resistance of *K. pneumoniae* Strains: (**a**) The growth curves of different *K. pneumoniae* strains over time. The x-axis shows time in hours (from 0 to 7), and the y-axis shows OD620, which ranges from 0 to 0.8. (**b**) The biofilm formation abilities of different bacterial strains. The x-axis lists the bacterial strains and control, while the y-axis shows OD550 values ranging from 0 to 0.8. ****, *p* < 0.0001; **, *p* < 0.01. (**c**) The survival percentages of different bacterial strains over time when exposed to human serum. The x-axis represents time in hours (from 0 to 3), and the y-axis shows the percentage of bacterial survival (ranging from 0% to 120%).

Growth curves and biofilm formation assays revealed variability among strains, but NJGLYY4165 did not exhibit a consistently distinct pattern compared with the reference panel (Figure 1a,b). Its biofilm-forming capacity was significantly higher than that of ATCCBAA1706 (*p* < 0.0001), but lower than that of ATCC2146 (*p* < 0.01) and ATCC13439 (*p* < 0.0001), and did not differ significantly from ATCC2524 or ATCCBAA1705 (*p* > 0.05). Overall, biofilm production by NJGLYY4165 fell within the range observed among commonly used reference strains.

In contrast, NJGLYY4165 demonstrated enhanced survival following exposure to normal human serum. After 3 h of incubation, this isolate exhibited the highest survival rate among the tested strains (Figure 1c), indicating strong resistance to complement-mediated killing under the experimental conditions.

**Figure 2:**
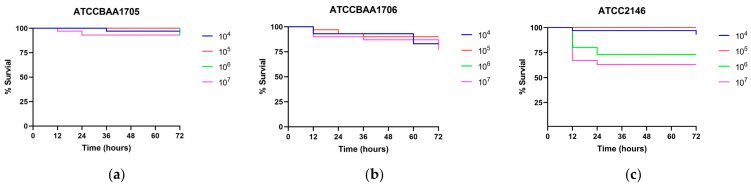

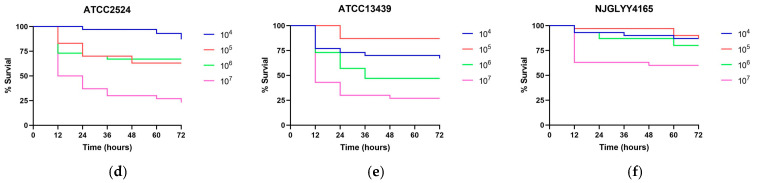
The survival rates of *G. mellonella larvae* infected with different concentrations of *K. pneumoniae* strains. The x-axis represents time in hours (from 0 to 72), and the y-axis represents the percentage of survival. (**a**), ATCCBAA1705; (**b**), ATCCBAA1706; (**c**), ATCC2146; (**d**), ATCC2524; (**e**), ATCC13439; (**f**), NJGLYY4165.

Virulence was further evaluated using the *Galleria mellonella* infection model. All reference strains induced dose-dependent lethality (Figure 2a–e). Notably, NJGLYY4165 displayed reduced lethality in this model: more than 50% of larvae remained viable at 48 h even at the highest inoculum, and mortality progressed more slowly compared with several reference strains (Figure 2f).

Collectively, these findings reveal a discordant phenotype in NJGLYY4165: despite pronounced resistance to complement-mediated killing in vitro, this isolate does not exhibit enhanced acute virulence in the invertebrate infection model. As the *G. mellonella* system does not recapitulate the human complement cascade, these results suggest that complement resistance may confer a context-dependent advantage that is not reflected in this model.

### 3.2. Host Inflammatory Responses Induced by NJGLYY4165 Are Comparable to Those Triggered by Reference Strains

To evaluate host inflammatory activation, IL-6 and IL-8 production were measured in epithelial (Int407) and macrophage (J774A.1) cell models following infection.

**Figure 3:**
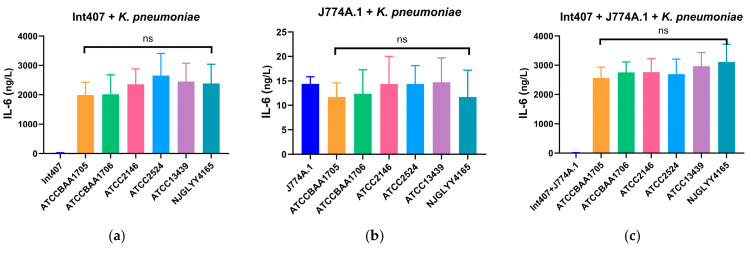

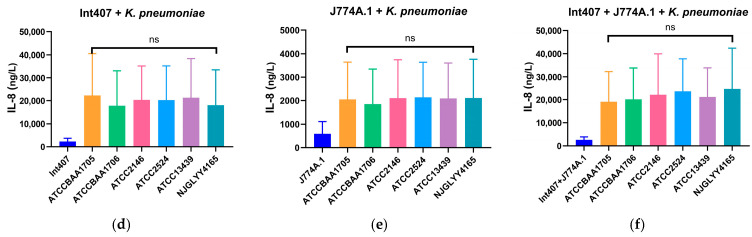
The levels of interleukin-6 (IL-6) and interleukin-8 (IL-8) produced by intestinal epithelial cells (Int407), murine macrophages (J774A.1), and their combination after infection with different strains of *K. pneumoniae*. The x-axis lists the bacterial strains, along with a control group, and the y-axis shows the IL-6 or IL-8 concentration in ng/L. ns, no significant difference. IL-6 Production (Panels (**a**–**c**)): (**a**) Int407 + *K. pneumoniae*; (**b**) J774A.1 + *K. pneumoniae;* (**c**) Int407 + J774A.1 + *K. pneumoniae*. IL-8 Production (Panels (**d**–**f**)): (**d**) Int407 + *K. pneumoniae*; (**e**) J774A.1 + *K. pneumoniae*; (**f**) Int407 + J774A.1 + *K. pneumoniae*.

In Int407 cells, infection with *K. pneumoniae* strains significantly increased IL-6 production compared with uninfected controls (Figure 3a). Similarly, IL-6 levels were elevated in infected J774A.1 macrophages, although the magnitude of induction was lower than that observed in epithelial cells (Figure 3b). Co-culture of Int407 and J774A.1 cells resulted in higher IL-6 levels compared with monocultures (Figure 3c). However, no consistent or strain-specific differences in IL-6 induction were observed between NJGLYY4165 and the reference strains.

A comparable pattern was observed for IL-8 production. Infection significantly increased IL-8 levels in both epithelial and macrophage cells relative to uninfected controls (Figure 3d,e). Co-culture conditions did not further enhance IL-8 production (Figure 3f), and again, no substantial strain-dependent differences were detected.

Overall, cytokine induction reflected a conserved host response to *K. pneumoniae* infection rather than a strain-specific inflammatory signature of NJGLYY4165.

### 3.3. Host Cell Interaction Is Associated with Extensive Transcriptional Changes in NJGLYY4165

To investigate bacterial transcriptional responses during host interaction, RNA sequencing was performed on NJGLYY4165 following 4 h co-culture with Int407 epithelial cells or J774A.1 macrophages.

**Figure 4:**
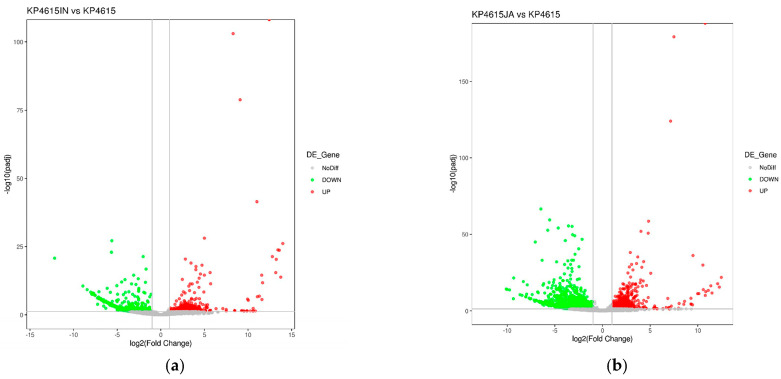
Significantly differentially expressed genes of KEGG pathway enrichment among *K. pneumoniae* ST11 (NJGLYY4615) during the interaction between intestinal cell (Int407) or macrophage (J774A.1). (**a**) ST11 vs. Int407 interaction; (**b**) ST11 vs. J774A.1 interaction. Volcano Plot of Significantly Differentially Expressed Genes. The X-axis represents the log2 fold change in gene expression between the two conditions. The Y-axis represents the negative log10 of the *p*-value, which indicates statistical significance. A higher value suggests a more significant difference in gene expression. Red dots represent genes with upregulated expression in the experimental condition (likely associated with the interaction of *K. pneumoniae* ST11 and Int407 cells or J774A.1 cells). Green dots represent genes with downregulated expression under the same condition. Gray dots represent genes with no significant differential expression between the two conditions.

Principal component analysis revealed clear separation between bacteria cultured under control conditions and those exposed to host cells (Figure 4), indicating that host cell exposure was associated with marked transcriptional variation at the examined time point.

Differential expression analysis identified 1043 significantly regulated genes during interaction with epithelial cells and 2116 genes during interaction with macrophages (Figure 4a,b). The greater number of differentially expressed genes under macrophage-associated conditions indicates a broader transcriptional response in the context of phagocytic cell exposure.

These findings demonstrate that NJGLYY4165 exhibits extensive transcriptional remodeling following host cell interaction at 4 h post-infection.

### 3.4. Central Metabolic Pathways Are Upregulated During Host Cell Exposure in NJGLYY4165

Functional enrichment analysis of upregulated genes revealed significant enrichment of metabolic pathways under host-associated conditions at the analyzed time point.

**Figure 5:**
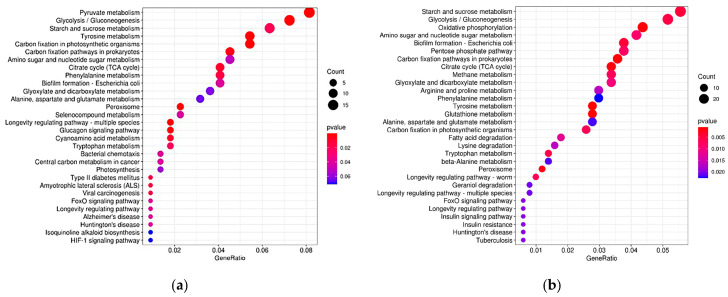
Significantly differentially bubble plot of KEGG pathway enrichment among *K. pneumoniae* ST11 (NJGLYY4615) during the interaction between intestinal cell (Int407) or macrophage (J774A.1). (**a**) Bubble plot of KEGG pathway enrichment for the differentially expressed genes of *K. pneumoniae* ST11 interacting with Int407 cells. (**b**) Bubble plot of KEGG pathway enrichment for the differentially expressed genes of *K. pneumoniae* ST11 interacting with J774A.1 cells. The Y-axis lists various KEGG pathways that are significantly enriched for differentially expressed genes. The X-axis represents the Gene Ratio, which indicates the proportion of genes in each pathway that are differentially expressed. Bubble size reflects the count of differentially expressed genes involved in each pathway, with larger bubbles indicating more genes involved. Color represents the *p*-value of the enrichment, where a redder color indicates a more statistically significant pathway (lower *p*-value).

In both epithelial and macrophage co-culture systems, genes involved in glycolysis/gluconeogenesis, pyruvate metabolism, and starch and sucrose metabolism were significantly enriched (Figure 5a,b). Under macrophage-associated conditions, additional enrichment was observed for the citrate cycle (TCA cycle), oxidative phosphorylation, and the pentose phosphate pathway (Figure 5b).

The concurrent upregulation of central carbon metabolism and energy-generating pathways indicates coordinated metabolic pathway activation during host cell exposure. These changes may reflect altered energetic and biosynthetic requirements under host-associated conditions, although temporal dynamics cannot be inferred from a single transcriptomic time point.

### 3.5. Upregulation of Iron Acquisition and ABC Transporter Systems During Host Interaction

**Figure 6:**
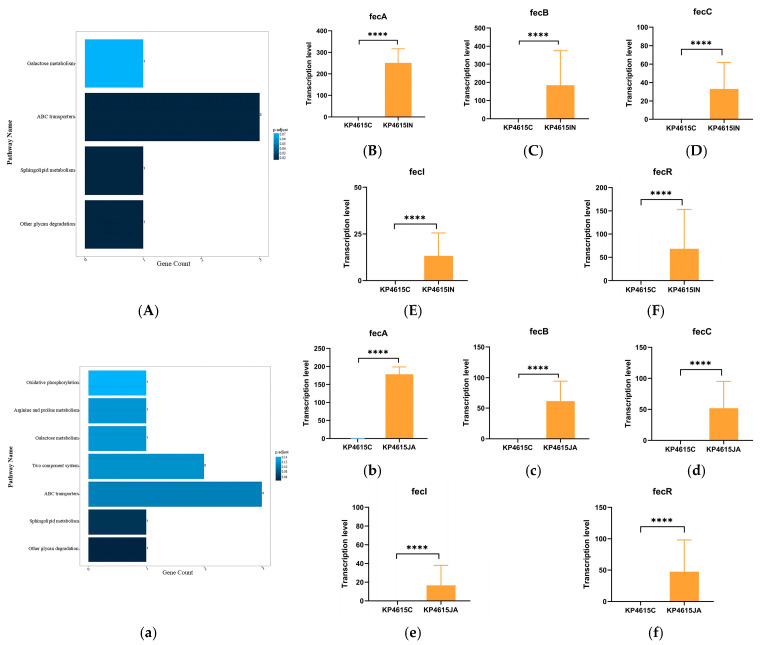
The dominant KEGG pathway enrichment based on the top 30 differentially expressed genes of KP4615 after interaction with host cells, and the predominant gene involved in the ATB transporter. Panel (**A**–**F**) The dominant KEGG pathway enrichment of KP4615 after interaction with intestinal epithelial cells Int407 and the predominant gene involved in ATB transporter; Panel (**a**–**f**) The dominant KEGG pathway enrichment of KP4615 after interaction with macrophages J774A.1 and the predominant gene involved in ATB transporter. ****, *p* < 0.0001.

ABC transporter systems were significantly enriched following host cell exposure (Figure 6A). Notably, genes associated with ferric citrate uptake—including *fecA*, *fecB*, *fecC*, *fecI*, and *fecR*—were upregulated during interaction with both epithelial cells and macrophages, with more pronounced induction under macrophage-associated conditions (Figure 6B–F).

Given the importance of iron acquisition for bacterial survival under host-imposed nutritional limitation, increased expression of the *fec* operon at this time point suggests enhanced iron uptake capacity during host interaction.

As this transcriptional analysis was conducted in a single ST11 isolate and at a single time point, without inclusion of additional epidemic or non-epidemic comparators, it remains unclear whether these responses represent isolate-specific features, lineage-associated characteristics, or broader species-level host response patterns.

## 4. Discussion

CRKP, particularly ST11 isolates, represents a major challenge for modern healthcare systems due to its widespread dissemination and multidrug resistance [20]. While previous studies have largely focused on antimicrobial resistance determinants and classical virulence factors [21], increasing evidence suggests that bacterial fitness traits—such as stress tolerance, immune evasion, and metabolic adaptability—may also contribute to persistence in hospital environments [22]. In this study, we characterized the clinical ST11 isolate NJGLYY4165 and identified a phenotype marked by strong serum resistance but without enhanced virulence or inflammatory activation under the experimental conditions tested.

A central finding of this work is the apparent dissociation between serum resistance and overt virulence. NJGLYY4165 demonstrated the highest survival following exposure to normal human serum, yet it exhibited attenuated lethality in the *Galleria mellonella* infection model compared with several reference strains. Serum resistance is generally considered an important determinant of bloodstream survival, reflecting the capacity to withstand complement-mediated killing [23]. However, our data indicate that enhanced complement tolerance does not necessarily translate into increased acute virulence. This observation underscores the multifactorial nature of pathogenicity and highlights that traits promoting survival under immune stress may be functionally distinct from those driving rapid host damage [24]. It should be noted that the *Galleria mellonella* model primarily reflects innate immune interactions and does not reproduce the human complement cascade or adaptive immune responses [25]. Therefore, this model cannot directly evaluate the contribution of complement resistance to bloodstream infection in humans.

Importantly, with the inclusion of statistical analysis, NJGLYY4165 did not exhibit significant differences in IL-6 or IL-8 induction or in biofilm formation compared with the tested reference strains. Thus, aside from its enhanced serum survival, the isolate appears phenotypically comparable under the experimental parameters applied. These findings suggest that, within the scope of the assays performed, NJGLYY4165 does not display exaggerated classical virulence traits that would directly explain epidemic success. Rather than hypervirulence, its phenotype may reflect enhanced tolerance to specific host-associated stress conditions.

The serum killing assay revealed a biphasic survival pattern characterized by an initial rapid reduction in bacterial counts followed by stabilization of a surviving subpopulation. The early decline likely reflects complement-mediated killing of serum-sensitive cells, whereas the subsequent plateau suggests persistence of a more tolerant fraction [26]. Such biphasic dynamics may arise from phenotypic heterogeneity within the population, including capsule-mediated surface protection, outer membrane remodeling, stress response activation, or persister-like states [27]. Although the present study does not experimentally dissect these mechanisms, the observed survival pattern is consistent with differential complement tolerance within the population rather than uniformly increased virulence [28].

From an ecological perspective, hospital-associated pathogens are continually exposed to antimicrobial pressure, host immune surveillance, and fluctuating nutrient availability [29,30]. Under these conditions, traits that enhance endurance and stress adaptation may facilitate persistence and transmission even in the absence of increased virulence [31,32]. The combination of strong serum resistance and reduced lethality observed in NJGLYY4165 is consistent with a phenotype oriented toward survival under immune pressure rather than aggressive host damage [33]. While the present data do not establish a direct link between this phenotype and epidemiological success, they support the broader notion that epidemic dissemination may not always correlate with elevated acute virulence [34]. However, because this study was conducted on a single isolate, it remains uncertain whether this phenotype reflects a broader feature of ST11 strains or represents isolate-specific characteristics.

At the transcriptional level, NJGLYY4165 displayed coordinated regulation of central metabolic pathways during host cell interaction at the examined 4 h time point. Enrichment of glycolysis, the tricarboxylic acid cycle, oxidative phosphorylation, and the pentose phosphate pathway indicates activation of core metabolic processes under host-associated conditions [35]. Similar transcriptional patterns have been reported in other multidrug-resistant pathogens exposed to environmental stress [36,37], suggesting that such responses may represent common host-associated transcriptional programs rather than lineage-specific traits. Because transcriptomic profiling was performed at a single time point, the present data represent a snapshot of gene expression changes and do not capture temporal transitions between early adaptation and longer-term persistence states.

Iron acquisition emerged as a prominent transcriptional feature. Host-mediated iron sequestration is a key component of nutritional immunity, and efficient iron uptake is critical for bacterial survival in vivo [38]. Upregulation of genes encoding the ferric citrate ABC transporter system during host interaction suggests increased expression of iron uptake machinery under iron-limited conditions [39]. Upregulation of genes encoding the ferric citrate ABC transporter system during host interaction suggests increased expression of iron uptake machinery under iron-limited conditions in vivo fitness [40].

Enrichment of additional ABC transporter systems further highlights the importance of nutrient acquisition and stress mitigation during host exposure [41]. Together with metabolic pathway activation, these findings indicate substantial transcriptional remodeling under host-associated conditions [42]. However, in the absence of comparative transcriptomic analyses across multiple lineages or time-course experiments, these results should be interpreted as descriptive observations of gene expression changes rather than evidence of dynamic metabolic flexibility or lineage-specific adaptation.

Several limitations should be acknowledged. First, this study is based on a single clinical ST11 CRKP isolate and does not capture the genetic diversity of the ST11 lineage. Second, apart from enhanced serum resistance, NJGLYY4165 did not display pronounced phenotypic differences compared with the reference strains in cytokine induction, biofilm formation, or the *Galleria mellonella* infection model. Third, although the *Galleria mellonella* model provides a convenient in vivo system, it does not replicate complement-dependent systemic infection or adaptive immune responses, limiting direct inference regarding bloodstream infection in humans. Fourth, the use of standard ATCC strains rather than contemporary non-ST11 clinical isolates restricts conclusions regarding lineage specificity. Finally, the transcriptomic analysis was performed at a single time point (4 h post-infection) and therefore does not allow assessment of temporal shifts in gene expression between early and late stages of host interaction. Time-course transcriptomic studies and functional validation will be required to clarify the dynamics and biological significance of the observed responses.

## 5. Conclusions

In conclusion, NJGLYY4165 represents a serum-resistant but non-hypervirulent ST11 CRKP isolate that illustrates a dissociation between complement tolerance and overt virulence under the experimental conditions tested. Although the present findings do not directly explain the epidemiological success of ST11, they provide a descriptive phenotypic and single-time-point transcriptomic characterization of host-associated responses in this isolate. Further comparative, time-course, and functional studies across diverse clinical strains will be necessary to determine the extent to which these responses are conserved and how they relate to lineage-level dissemination and clinical outcomes.

## Data Availability

The data presented in this study are available in the NCBI Sequence Read Archive (SRA) under BioProject number PRJNA1180697. The records can be accessed at https://www.ncbi.nlm.nih.gov/sra/PRJNA1180697 (accessed on 1 March 2026).

## Abbreviations

The following abbreviations are used in this manuscript:

CRKP	Carbapenem-resistant *Klebsiella pneumoniae*
ST11	Sequence type 11
ATCC	American Type Culture Collection
MALDI-TOF MS	Matrix-Assisted Laser Desorption/Ionization Time-of-Flight Mass Spectrometry
LB	Lysogeny Broth
WGS	Whole-genome sequencing
PBS	Phosphate-buffered saline
EMEM	Eagle’s Minimum Essential Medium
DMEM	Dulbecco’s Modified Eagle Medium
MOI	Multiplicity of infection
IL-6	Interleukin-6
IL-8	Interleukin-8
GO	Gene Ontology
KEGG	Kyoto Encyclopedia of Genes and Genomes
SD	Standard deviation
ANOVA	One-way analysis of variance
TCA cycle	The citrate cycle
ABC transporter	ATP-binding cassette transporter
